# The effect of structured medication review followed by face-to-face feedback to prescribers on adverse drug events recognition and prevention in older inpatients – a multicenter interrupted time series study

**DOI:** 10.1186/s12877-022-03118-z

**Published:** 2022-06-17

**Authors:** Joanna E. Klopotowska, Paul F. M. Kuks, Peter C. Wierenga, Clementine C. M. Stuijt, Lambertus Arisz, Marcel G. W. Dijkgraaf, Nicolette de Keizer, Susanne M. Smorenburg, Sophia E. de Rooij, on behalf of the WINGS Study Group, Joost L. B. Hoekstra, Minke E. P. Jansen, Wim G. Meijer, Bea M. van der Kleij, Anne M. Lagaay, Ruud T. M. van der Hoeven

**Affiliations:** 1 Amsterdam University Medical Centers location University of Amsterdam, Medical Informatics, Amsterdam, The Netherlands; 2 Amsterdam Public Health, Quality of Care, Amsterdam, The Netherlands; 3 Amsterdam University Medical Centers location University of Amsterdam, Pharmacy and Clinical Pharmacology, Amsterdam, The Netherlands; 4 Gelderse Vallei Hospital, Hospital Pharmacy, Ede, The Netherlands; 5 Center of Excellence on Parkinson’s disease (Punt voor Parkinson), Groningen, The Netherlands; 6 Amsterdam University Medical Centers location University of Amsterdam, Internal Medicine, Amsterdam, The Netherlands; 7 Amsterdam University Medical Centers location University of Amsterdam, Epidemiology and Data Science, Amsterdam, The Netherlands; 8 Amsterdam Public Health, Methodology, Amsterdam, the Netherlands; 9 Amstelland Hospital, Board of Directors, Amstelveen, The Netherlands

**Keywords:** Adverse drug events, Elderly, Inpatients, Medication review, Clinical pharmacist, Interrupted time series

## Abstract

**Background:**

The effectiveness of interventions to improve medication safety in older inpatients is unclear, given a paucity of properly designed intervention studies applying clinically relevant endpoints such as hospital-acquired preventable Adverse Drug Events (pADEs) and unrecognized Adverse Drug Events (uADEs). Therefore, we conducted a quality improvement study and used hospital-acquired pADEs and uADEs as main outcomes to assess the effect of an intervention aimed to improve medication safety in older inpatients.

**Method:**

The study followed an interrupted time series design and consisted of three equally spaced sampling points during baseline and during intervention measurements. Each sampling point included between 80 to 90 patients. A total of 500 inpatients ≥65 years and admitted to internal medicine wards of three Dutch hospitals were included. An expert team retrospectively identified and assessed ADEs via a structured patient chart review. The findings from baseline measurement and meetings with the internal medicine and hospital pharmacy staff were used to design the intervention. The intervention consisted of a structured medication review by hospital pharmacists, followed by face-to-face feedback to prescribers, on average 3 days per week.

**Results:**

The rate of hospital-acquired pADEs per 100 hospitalizations was reduced by 50.6% (difference 16.8, 95% confidence interval (CI): 9.0 to 24.6, *P* <  0.001), serious hospital-acquired pADEs by 62.7% (difference 12.8, 95% CI: 6.4 to 19.2, *P* <  0.001), and uADEs by 51.8% (difference 11.2, 95% CI: 4.4 to 18.0, *P* <  0.001). Additional analyses confirmed the robustness of the intervention effect, but residual bias cannot be excluded.

**Conclusions:**

The intervention significantly decreased the overall and serious hospital-acquired pADE occurrence in older inpatients, and significantly improved overall ADE recognition by prescribers.

**Trial registration:**

International Standard Randomized Controlled Trial Number Register, trial registration number: ISRCTN64974377, registration date (date assigned): 07/02/2011.

**Supplementary Information:**

The online version contains supplementary material available at 10.1186/s12877-022-03118-z.

## Background

Adverse drugs events (ADEs) are one of the most common adverse events in all healthcare settings [1]. An ADE is usually defined as any harmful event resulting from drug therapy. ADEs include adverse drug reactions (ADRs) resulting from appropriate care and causing any degree of non-preventable patient harm (i.e. drug side-effects), as well as preventable ADEs (pADEs) resulting from a medication error (omission or commission) and causing any degree of preventable patient harm [1, 2]. ADEs are associated with a prolonged hospital stay, a two-fold increase in the risk of death, and higher hospital costs [3, 4].

Older patients are especially at risk for ADEs due to multimorbidity, polypharmacy, cognitive decline, and altered physiological functions [5]. Preventable ADEs that occur during hospitalization, i.e. hospital-acquired pADEs, are among the most serious medication safety risks in older inpatients, with prescribing errors as the primary cause [6–8]. In addition, an atypical disease presentation in older patients is frequent and may leave ADEs unrecognized by physicians (uADEs) [9, 10]. For these reasons, improving safety of medication prescribing in these, often vulnerable and complex, patients has become a major patient safety goal in hospitals [5, 11].

Yet, the effectiveness of interventions aiming to improve safety of medication prescribing in older inpatients remains controversial, since most studies describing such interventions used surrogate endpoints, such as prescription errors, “medication appropriateness” or “medication-related problems” [12–20]. There is a paucity of properly designed intervention studies in older inpatients applying clinical endpoints such as the incidence and severity of hospital-acquired pADEs, or hospital readmissions related to preventable ADEs, endpoints that are directly related to medication use and knowledge [12–20]. Hospital-acquired pADEs seem to be more appropriate than generic clinical endpoints such as length of stay, all-cause mortality, and all-cause hospital readmission, since hospital-acquired pADEs measure aspects which may be directly affected (i.e. causally linked) by in-hospital interventions aiming to manage risks of prescribed drugs and to reduce drug-related harm [13, 16]. To our knowledge, uADEs have not been used previously to assess the effect of prescribing safety interventions.

In previous studies, we found that 71% of hospital-acquired ADEs in older inpatients were preventable because they were caused by prescribing errors, and 20% of ADEs (community- or hospital-acquired ADEs) failed to be recognized (uADEs) by the medical teams involved in patient care during hospital stay [19, 20]. These results prompted us to design an intervention aiming at improvement of prescribing safety by involving the staff and residents of internal medicine and hospital pharmacy departments. We felt that involving frontline workers and tailoring intervention to local needs and resources were crucial to the implementation success of any prescribing safety intervention [21–23]. Here, we present the effect of our intervention on hospital-acquired pADEs, hospital-acquired serious pADEs and uADEs (community- or hospital-acquired) in older inpatients.

## Methods

The study protocol has been published elsewhere [24]. This study is reported according to Revised Standards for Quality Improvement Reporting Excellence SQUIRE 2.0 [25]. The reporting checklist can be found as Additional file 1. Furthermore, regarding the context, intervention development and intervention implementation, here only essential information is provided. A more detailed description of these elements can be found in Additional file 2 presented according to The TIDieR (Template for Intervention Description and Replication) Checklist [26].

### Context

The study was conducted in one academic and two non-academic hospitals in The Netherlands. The intervention was delivered to Internal Medicine wards of the participating hospitals. In the Netherlands (as well as in most European countries), hospital pharmacists provide only limited supervision on prescribing and are not part of medical teams on the wards [27]. This is different from other countries such as the United Kingdom (UK) and the United States (US) of America, employing more pharmacists per bed, and providing pharmaceutical care under the denominator “clinical pharmacy” [28, 29]. Pharmacists in most European countries, except the UK, are less numerous, and tend to be generalists with broader, but more superficial, pharmacotherapy expertise [27].

The daily care of patients on the Internal Medicine wards in the participating hospitals was provided by junior medical residents, who had one to two years of clinical experience and were supervised by attending senior physicians. Gaps in geriatric pharmacotherapy knowledge and skills were felt to be of major concern across all care settings and levels of medical experience [30].

### Intervention

To develop the intervention, multidisciplinary meetings (physicians and pharmacists) were organized at which the Bow-Tie model was used to structure the discussion about causes, errors, preventive and recovery measures [31, 32], in relation to the ADE results from the baseline measurement. Based on the Bow-Tie analyses, the intervention ultimately implemented consisted of a medication review and face-to-face feedback on prescribing by a hospital pharmacist, on average three days per week. The medication reviews were conducted from 1 October 2009 to 30 June 2010. All (potential) DRPs identified, together with the recommendations to resolve these, were registered on a standardized consultation form. Subsequently, the results of the medication review were discussed face-to-face with the internal medicine residents on the wards. Such face-to-face discussions facilitated the exchange of knowledge and any additional information about the patient’s condition. The hospital pharmacist recorded whether proposed recommendations were accepted by the internal medicine residents or not.

### Study of the intervention

We conducted a multicenter interrupted time series (ITS) study between 1 April 2007 and 30 June 2010 in the Internal Medicine wards of the participating hospitals. The ITS design followed the Cochrane Effective Practice and Organization of Care Review Group (EPOC) criteria for short time series [33]. An ITS design is a well-accepted quasi-experimental approach for evaluating interventions at the health system level, in which randomization or identification of a control group is often not feasible [34, 35]. The ITS sampling strategy of this study consisted of three evenly spaced sampling points during the baseline measurement (consisting of eight months) and three evenly spaced sampling points during the intervention measurement (also consisting of eight months and starting one month after the introduction of the intervention). The number of patients included in each hospital was equal across measurement periods and sampling points. A visualization of our sampling strategy is available as Additional file 3.

Sample size and power calculations, using results from Leape et al. [28] as guidance, showed that based on the expected incidence of 15 preventable ADEs per 100 hospitalizations, 496 patient admissions were needed, equally divided between the pre- and post-intervention periods [α = 0.05 and β = 0.8]. A Poisson distribution was assumed for preventable ADEs, to detect a clinically relevant and statistically significant reduction of 7.5 preventable ADEs per 100 hospitalizations (50%) by our intervention. This total number of 496 patient admissions fulfilled the EPOC criteria of at least 180 observations equally divided between pre- and post-measurement, and having at least three data points in pre-measurement and in post-measurement, with at least 30 observations per data point [33].

### Main outcome measures

We assessed the effect of the intervention on three clinical outcomes: 1) the change in the rate of hospital-acquired pADEs, 2) the change in the rate of serious hospital-acquired pADEs, and 3) the change in the rate of uADEs. For calculating the rate of uADEs (consisting of unrecognized pADEs and unrecognized ADRs), both hospital-acquired and community-acquired uADEs were considered. The primary outcome measure was the rate of hospital-acquired pADEs. The secondary outcomes were the rate of serious hospital-acquired pADEs and the rate of uADEs. All outcomes were calculated as rates per 100 hospitalizations.

As an internal validity measure of our study, we assessed the change in the rate of ADRs. ADRs are not reducible by definition, because they are usually considered not preventable. Therefore, their rate is independent of any intervention and expected to remain constant during the whole study period. A shift in the number of ADRs could point to an inconsistent ADE review process. For calculating the rate of ADRs, both hospital-acquired and community-acquired ADRs were considered, and expressed per 100 hospitalizations.

These outcomes differ in four ways from the ones described in our study protocol [24]. First, we did measure ADEs at admission (i.e. ADEs which occurred before hospitalization) and assessed their severity and preventability. However, since ADEs already present at admission can impossibly be reduced by an intervention during hospitalization, we did not use ADEs at admission for the evaluation of our intervention. Instead, we assessed if the internal medicine physicians recognized these admission ADEs during hospitalization of the older inpatients, and incorporated these findings in our uADE outcome (which includes the number of recognized ADEs at admission mentioned in the study protocol). Second, the serious hospital-acquired pADEs and ADRs as outcome measures were added to test the robustness of the intervention effect. Third, we measured medication errors but we did not use the number of medication errors per number of medication orders as a secondary outcome measure, because the number of medication errors is reflected in the hospital-acquired pADE rate. Lastly, we did not include the number of readmissions within three months after the index hospitalisation because, as pointed out in the introduction, this outcome is too generic and not well causally linked to our intervention. To evaluate the intervention on the process level, the acceptance rate of all hospital pharmacist recommendations was calculated [13, 16].

ADE definitions used in this study align with internationally accepted definitions and are consistent with definitions used in previous studies [1, 2, 12–18]. An *ADE* was defined as any harmful event resulting from drug therapy – from appropriate care (*ADR*), or inappropriate care (*pADE*). A *serious pADE* was defined as a pADE causing severe (grade 3), life-threatening (grade 4), or fatal (grade 5) preventable patient harm according to the Common Terminology Criteria for Adverse Events version 3.0 (CTCAEv3), [36]. Grades 3 to 5 of the CTCAEv3 correspond to the definition of a serious ADE by the World Health Organization (WHO: harm resulting in death, harm requiring inpatient hospitalization or prolongation of existing hospitalization, harm resulting in persistent or significant disability/incapacity, or life-threatening harm), [2]. An *unrecognized ADE* was a pADE or ADR identified by the ADE-identifying expert team (see Assessment of ADEs) but not identified by the prescriber. *Hospital-acquired* means an ADE occurred during hospital stay. *Community-acquired* means an ADE was present upon admission but occurred in home setting.

Data on the occurrence of ADEs were collected from cohorts of consecutively admitted patients of 65 years and older admitted to one of the internal medicine wards of the participating hospitals. Apart from age, the other inclusion criterion was the use of five or more medications at admission. Patients were included only once during the whole study period (index hospitalization). For the baseline measurement, all eligible and consecutively admitted patients between April 2007 and 30 November 2007 were included. For the intervention measurement, all eligible and consecutively admitted patients between November 2009 and 30 June 2010 were included. Patients on chemotherapy, radiation therapy, or stem cell/kidney transplantation were excluded, as well as patients discharged within 24 hours and patients which had been transferred from other hospitals or other non-medical wards within the study hospitals.

### ADE identification and assessment

The process of ADE identification and assessment is described in detail elsewhere and showed good reliability [19]. In short, the complete medical records of the included patients were first abstracted by trained study assistants. This abstraction took place between September 2007 and April 2008 for the patients included during the baseline measurement period, and between December 2009 and September 2010 for the patients included during the intervention period.

Subsequently, all information abstracted was presented for a systematic patient chart review by an independent team of two clinical experts: a senior medical specialist in internal medicine (LA), and a clinical pharmacist expert in geriatric pharmacotherapy (CS). Both experts were well acquainted with patient chart review methodology and ADE assessment. This expert team remained unchanged throughout the entire study. The experts were not blinded to the status of patients’ charts (baseline or intervention period). This was a deliberate choice, because in order to develop our intervention, we needed to know the extent and type of ADEs from the baseline measurement. An awareness bias, because the experts were not blinded to the period, was limited by involving experts who did not participate in the intervention or the daily care of patients in the participating hospitals.

These reviews took place between June 2008 and February 2009 for patients included in the baseline measurement, and between January 2010 and January 2011 for patients included in the intervention measurement. The causality was assessed according to an adapted version of the WHO – Uppsala Medical Centre criteria [37]. Only ADEs assessed as having possible, probable, or nearly certain causality with drug commission or omission were included. The severity of ADEs was assessed according to CTCAEv3 criteria [36]. ADEs were judged to be preventable if they were caused by a medication error, as assessed using prevailing national and local pharmacotherapy standards [38].

### Analyses

We compared the baseline and intervention periods and adjusted the effect of the intervention on our primary and secondary outcome measures for background trends over time, and for other potential confounders. For that purpose, we applied generalized linear modeling with Poisson link functions [34, 39]. First, level and/or trend changes in hospital-acquired pADEs and uADEs during the entire study period were analyzed. The following ITS parameters were included: the change in level post-intervention (baseline measurement coded as zero, intervention measurement coded as one), the pre-intervention trend (time according to data points sequence coded successively from the start of the baseline measurement; one to six), and post-intervention trend (time after intervention according to the data points sequence coded as zero before the start of the intervention and coded successively from the start of the intervention; one to three). Second, patient variables shown in Table 1 with a *P* value ≤0.1 as identified through univariate analyses as well as variables significantly different between patients included during the baseline and intervention measurements, were added to a multivariate model with significant ITS parameters, to account for confounding and verify the absence of any bias due to differences in the case mix between the baseline and the intervention period. Third, we removed non-significant variables (*P* value ≥0.05) through step-wise backward elimination. Our aim was to develop the most parsimonious model. Parsimonious models have optimal parsimony, or just the right amount of predictors needed to explain the model well (a model that neither under-fits nor over-fits). Step-wise backward elimination aligns with this goal.

**Table 1:** Characteristics of the baseline and intervention measurement cohorts

Characteristic	Baseline measurement (***n*** = 250)	Intervention measurement (*n* = 250)	*P *value^*^
**Age, mean in years ± SD in years**	76.9 ± 7.5	77.2 ± 7.9	0.655
**Female, n (%)**	133 (53.2)	124 (49.6)	0.421
**Living independently, n (%)**	211 (84.4)	196 (78.4)	0.085
**Type of admission: acute admission, n (%)**	213 (85.2)	223 (89.2)	0.181
**Time of admission: weekday admission, n (%)**	140 (56.0)	168 (67.2)	0.010
**Length of stay**^**a**^**, median; IQR**	5.9; 6.0	6.0; 8.1	0.778
**Number of preadmission medications, mean ± SD**	7.31 ± 3.2	7.85 ± 3.7	0.084
**Number of hospital medications, mean ± SD**	11.0 ± 4.1	12.7 ± 5.0	< 0.001
**Charlson Co-morbidity Index score, mean ± SD**	2.78 ± 2.0	2.87 ± 1.9	0.613
**Number of concomitant diseases, mean ± SD**	3.16 ± 1.7	3.39 ± 1.9	0.150
**Cognitive impairment on admission**^**b**^**, n (%)**	45 (18.0)	52 (20.8)	0.429
**MDRD** ***e*GFR**^**c**^ **(ml/min/1.73 m**^**2**^**), n (%)**	*n* = 240	*n* = 245	0.946
≥ 60	94 (39.2)	93 (38.0)	
30–59	89 (37.1)	91 (37.1)	
≤ 29	57 (23.8)	61 (24.9)	

_*SD* standard deviation, *IQR *interquartile range, *MDRD eGFR *Modification of Diet in Renal Disease estimated Glomerular Filtration Rate. *Test for standard *P* value across quantitative or ordinal quantitative variables._^a^_Length of stay on the Internal Medicine ward._^b^_Cognitive impairment on admission due to delirium, unconsciousness, general cognitive decline, drowsiness or psychiatric disease._^c^_MDRD *e*GFR on admission_

Descriptive statistics were applied for the analysis of patient characteristics, including means, standard deviations, medians, and interquartile ranges. To test for differences between patients included during the baseline and intervention measurement periods, categorical variables were analyzed using the chi-square test, and normally distributed continuous variables were analyzed using Student’s *t*-test for independent samples. Numerical variables were tested for normal distribution using the Kolmogorov–Smirnov test. Non-normally distributed continuous variables were analyzed using the Mann-Whitney *U* test. Computer software (SPSS versions 18.0 and 19.0, SPSS Inc., Chicago, IL, USA) was used for the computations.

## Results

### Study population

The characteristics of older inpatients included during the baseline and intervention measurements were comparable (Table 1). The two groups of patients differed in the number of hospital medications (*P* <  0.001) and weekday versus weekend admission (*P* = 0.01). We have explored the differences in patient characteristics per measurement period between the participating hospitals. No significant differences were identified. The results of the baseline measurement have been published previously [19], but are included here again to allow for easy comparison with the results of the intervention measurement.

### Main outcomes

The effect of the intervention on the primary and secondary outcomes are shown in Table 2. The rate of hospital-acquired pADEs per 100 hospitalizations declined by 50.6%, from 33.2 during the baseline to 16.4 during the intervention measurement (a rate difference of 16.8, 95% confidence interval (CI): 9.0 to 24.6, *P* <  0.001). The rate of serious hospital-acquired pADEs per 100 hospitalizations declined by 62.7%, from 20.4 during the baseline measurement to 7.6 during the intervention measurement (a rate difference of 12.8, 95% CI: 6.4 to 19.2, *P* <  0.001). The rate of uADEs per 100 hospitalizations declined by 51.8%, from 21.6 during the baseline measurement to 10.4 during the intervention measurement (a rate difference of 11.2, 95% CI: 4.4 to 18.0, P <  0.001). The rate of ADRs per 100 hospitalizations remained constant (difference 0.80, 95% CI: − 8.3 to 10.0, *P* = 0.86).

**Table 2:** The effect of the intervention

Outcome measures^**a**^	Baseline measurement (95% CI)	Intervention measurement (95% CI)	Rate difference (95% CI)	***P*** **value**
**All hospital-acquired pADEs**	33.2 (26.8 to 41.2)	16.4 (12.1 to 22.3)	16.8 (9.0 to 24.6)	< 0.001
**Serious hospital-acquired pADEs**	20.4 (15.5 to 26.8)	7.60 (4.9 to 11.9)	12.8 (6.4 to 19.2)	< 0.001
**uADEs**	21.6 (16.5 to 28.2)	10.4 (7.1 to 15.3)	11.2 (4.4 to 18.0)	< 0.001
**ADRs**	53.6 (45.3 to 63.5)	52.8 (44.5 to 62.6)	0.80 (−8.4 to 10.0)	0.86

*CI* confidence interval, *pADEs *preventable adverse drug events, *uADEs *unrecognized adverse drug events, *ADRs* adverse drug reactions. ^a^The outcome measures are expressed in rates per 100 hospitalizations with 95% CIs

No significant pre- or post-intervention trends were identified for hospital-acquired pADEs and uADEs. Therefore, only the ITS parameter “change in the level post-intervention” was included in the multivariate analyses. A visualization of a trend over time in hospital-acquired pADEs and in uADEs is available as Additional file 3. Our global ITS models for both outcomes are presented as Additional file 4. Regarding patient characteristics, variables with *P* value ≤0.1 as identified through univariate analyses or showing significant difference between baseline and intervention measurement patients, were taken into account when constructing the multivariate models. Outputs of univariate analyses are presented as Additional file 5. The final multivariate models without and with backward elimination are shown in Table 3. The odds ratio (OR) of experiencing a hospital-acquired pADE was nearly 60% lower during the intervention measurement in comparison to the baseline measurement (OR 0.40, 95% CI: 0.28 to 0.60, *P* < 0.001), and the OR of experiencing an uADE was 53% lower (OR 0.47, 95% CI: 0.29 to 0.75, *P* = 0.002).

**Table 3:** The final multivariate models for the effect of the intervention

	**Hospital-acquired pADE**^**a**^			
	**Multivariate model start**		**Multivariate model final**	
**Variables**	**OR (95% CI)**	***P*** **value**	**OR (95% CI)**	***P*** **value**
**Change in level post-intervention**				
Intervention period	0.42 (0.29 to 0.62)	0.009	0.40 (0.28 to 0.60)	< 0.001
Baseline period	RC		RC	
**Type of admission**			Out on 4th step	
Acute	0.64 (0.41 to 1.00)	0.051		
Elective	RC			
**Number of preadmission medications**	0.99 (0.93 to 1.05)	0.687	Out on 2nd step	
**Number of hospital medications**	1.09 (1.05 to 1.13)	< 0.001	1.09 (1.05 to 1.13)	< 0.001
**Charlson Co-morbidity Index score**	1.03 (0.93 to 1.13)	0.571	Out on 1st step	
**MDRD eGFR (ml/min/1.73 m**^**2**^**)**			Out on 3rd step	
≤ 29	1.52 (0.93 to 2.48)	0.097		
30–59	1.43 (0.93 to 2.21)	0.108		
≥ 60	RC			
	**uADEs**^**a**^			
	**Multivariate model start**		**Multivariate model final**^**b**^	
**Variables**	**OR (95% CI)**	***P*** **value**	**OR (95% CI)**	***P*** **value**
**Change in level post-intervention**				
Intervention	0.47 (0.29 to 0.75)	0.002	0.47 (0.29 to 0.75)	0.002
Baseline	RC		RC	
**Number of preadmission medications**	1.11 (1.04–1.18)	0.001	1.11 (1.04–1.18)	0.001
**Charlson Co-morbidity Index score**	0.85 (0.74–0.96)	0.012	0.85 (0.74–0.96)	0.012

*pADEs *preventable adverse drug events, *OR *odds ratio, *CI *confidence interval, *RC *reference category,* uADEs *unrecognized adverse drug events, *NA *not applicable, *MDRD eGFR *Modification of Diet in Renal Disease estimated Glomerular Filtration Rate. ^a^Because creatinine was not measured in 15 patients, the analyses presented in this table were conducted with 485 patients instead of all 500 patients included in the study. MDRD eGFR on admission. ^b^All predictors were retained. Therefore, the final model is the same as the starting model

### Types of identified hospital-acquired pADEs and uADEs

**Fig. 1:**
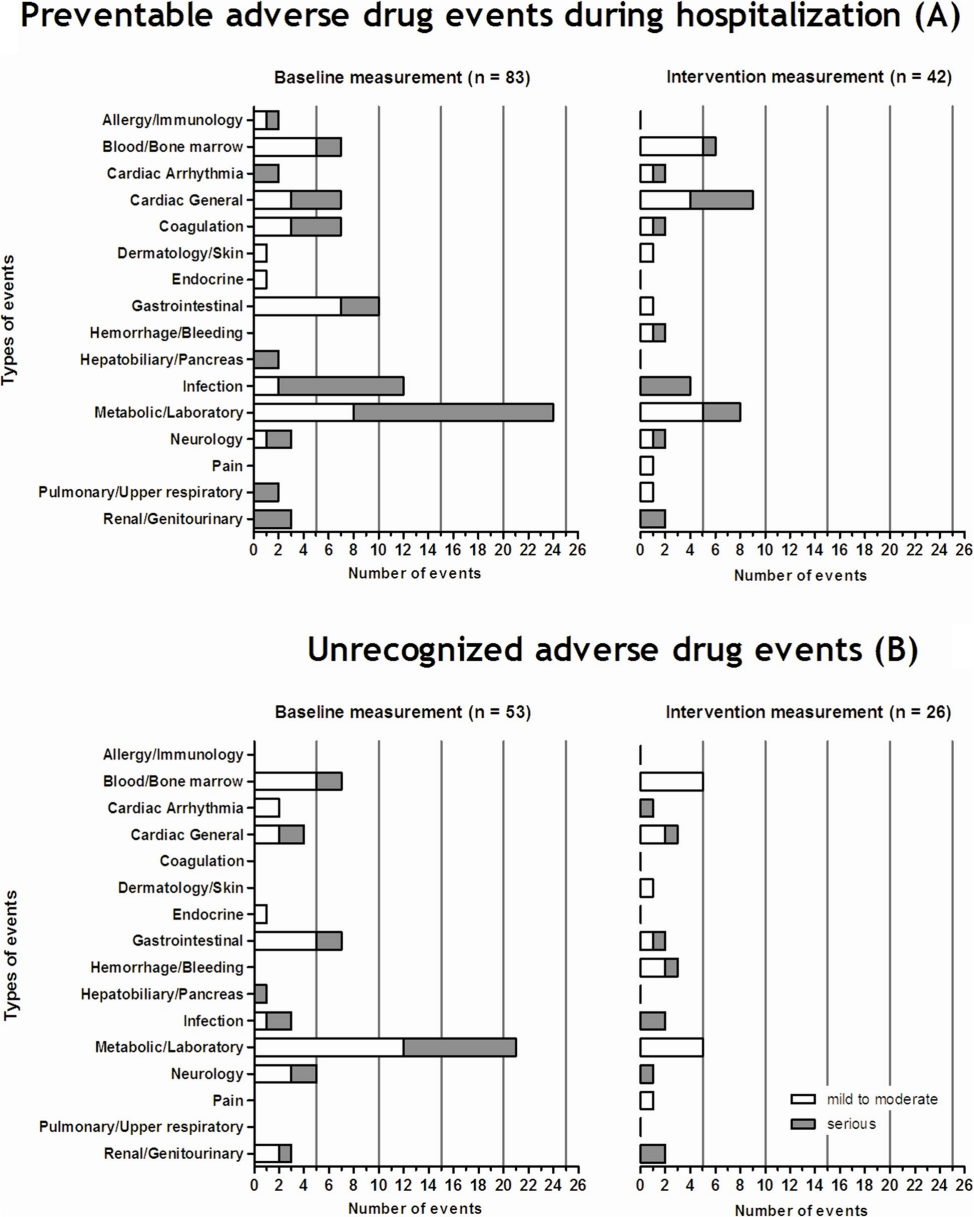
Types of hospital-acquired pADEs (**A**) and uADEs (**B**) identified during the baseline and intervention measurements. Mild to moderate hospital-acquired pADEs or uADEs correspond to grade 1 to 2 of the Common Terminology Criteria for Adverse Events criteria version 3.0 (CTCAEv3) [39]. Serious hospital-acquired pADEs or uADEs are adverse events, which caused severe, life-threatening, or fatal patient harm (grade 3 to 5 of the CTCAEv3)

The most common type of events related to hospital-acquired pADEs and uADEs during both measurement periods are shown in Fig. 1.

Of the blood/bone marrow-related hospital-acquired pADEs, 84.6% correspond to prolonged anemia due to omission of iron supplements mainly in patients with chronic cardiovascular disease who were hospitalized due to (excessive) blood loss. Of all cardiac general events, 62.5% were hypo- or hypertensive events, and all (100%) coagulation events were cases of an elevated INR beyond the upper limit of normal in patients taking a coumarine derivative. Constipation, diarrhea, nausea, or vomiting constituted the majority (81.0%) of gastrointestinal events. Infection-related hospital-acquired pADEs were mainly (93.8%) cases of an inappropriate empirical antibiotic therapy and/or route of administration, (harm outcome: delayed recovery or lack of clinical improvement). Of the metabolic/laboratory events, 82.8% were either hypo- or hyperkalemia, hypo- or hyperglycemia, elevated liver function tests, or raised creatinine values.

During the baseline measurement, 83 hospital-acquired pADEs were identified by the expert team, compared to 41 hospital-acquired pADEs during the intervention measurement. In total, 90.5% of this reduction was related to fewer metabolic/laboratory-related hospital-acquired pADEs, gastrointestinal-related hospital-acquired pADEs, coagulation-related hospital-acquired pADEs, and infection-related hospital-acquired pADEs. During the baseline measurement, 54 uADEs were identified, compared to 26 uADEs during the intervention measurement. In total, 89.3% of this reduction was related to fewer metabolic/laboratory-related uADEs, gastrointestinal-related uADEs, and neurological-related uADEs.

### Medication errors resulting in hospital-acquired pADEs

Most hospital-acquired pADEs during both measurement periods were related to prescribing errors (87.9%) of which under- and overtreatment (36.7%), prescribing contra-indicated medications (23.6%), and dosing errors (17.4%) were most common (Additional file 6). In total, 81.1% of the reduction in hospital-acquired pADE during the intervention period can be attributed to reduced use of contra-indicated medication, less dosing errors or inappropriate choice errors. Hospital-acquired pADEs due to under- and overtreatment appear to be least affected by the intervention.

### The acceptance of hospital pharmacists’ recommendations

During the intervention period, a total of 400 recommendations were made by hospital pharmacists (Additional file 7). Overall, 61.5% of these recommendations were accepted by the medical teams. Recommendations regarding “no clear indication” and “inappropriate choice of medication for an indication” were least accepted (38.9 and 8.3%, respectively).

## Discussion

A structured medication review and face-to-face feedback to internal medicine residents by hospital pharmacists resulted in a significant reduction of preventable medication-related harm in older inpatients (50.6% reduction in hospital-acquired pADEs and 62.7% reduction in serious hospital-acquired pADEs, both *P* < 0.001) and a significant improvement of ADE recognition by medical teams (51.8% reduction in uADEs, *P* < 0.001). In line with previously published studies [40, 41], our results show that hospital-acquired pADEs in older inpatients are common and mainly caused by prescribing errors, and ADEs (which consist of pADEs and ADRs) are often unrecognized (uADEs). This illustrates the need for an intervention to optimize safe prescribing as well as ADE recognition by medical teams. Because of the complexity of the medication prescribing process and the complexity of older inpatients’ cases, a systematic assessment and monitoring of pharmacotherapy may help reduce pADEs and uADEs [5, 42]. We found a higher rate of pADEs in comparison to what was expected based on our sample size and power calculation. This can be explained by the high sensitivity of our patient chart review method for ADEs [19], the high-risk for ADEs of our study population given older age and polypharmacy [5, 6], and the inclusion of all types of severity in our ADE definition.

To our knowledge, this is the first study in older inpatients presenting an evaluation of the effect of a medication review on uADEs, and one of only few studies on hospital-acquired pADEs [12–18]. In a recent systematic review by Beuscart and colleagues [13], it was found that only 9% of published trials (4 out of 47) that evaluated the impact of medication reviews, measured (serious) hospital-acquired ADEs as a primary or secondary outcome. Of these, only one study was conducted in older inpatients. The authors state that this gap in evidence could be explained by the complex and time-consuming nature of measuring ADEs via patient chart review. Our experiences in this study support this view. However, since our purpose was to evaluate an intervention aiming to reduce drug-related patient harm in a setting where insights on the extent and type of this harm were limited, measuring pADEs and uADEs (outcomes causally linked to the intervention) via an ADE-sensitive method like the patient chart review seemed most appropriate [13, 16].

From seven recently published systematic reviews on interventions to improve medication safety in older patients [12–18], we identified six published studies in which ADE-related measures were used as outcomes to evaluate the effect of medication review for older inpatients [43–48]. Schmader and colleagues [43] measured ADRs and found no change in all ADRs (*P* = 0.12) or serious ADRs (*P* = 0.41) after introducing a geriatric evaluation and management intervention for medical or surgical inpatients. In a study by Trivalle and colleagues [44], a physician and a nurse provided specific information during one week to a medical team about prescribing in older inpatients and how to identify and prevent ADEs. ADEs were reduced by 14% (*p* = 0.004) in the intervention group compared to the control group. O’Connor and colleagues [45] introduced the Screening Tool of Older Persons’ Prescriptions (STOPP) and the Screening Tool to Alert to Right Treatment (START) for inappropriate prescriptions and found the number of participants in the intervention group with definitely or possibly avoidable hospital-acquired ADRs was 48% lower in comparison to the control group. O’Sullivan and colleagues [46] found that a clinical decision support system (CDSS)-supported structured medication review by a pharmacist reduced the number of definitely or possibly avoidable hospital-acquired ADRs by 34%. In a study by Wehling and colleagues [47], the Fit fOR The Aged (FORTA) score was used as a tool to support medication review in older inpatients. Using FORTA, medications to treat chronic illnesses in older patients are labeled from indispensable, beneficial, questionable to avoid. They found a 20% reduction in all ADRs (*p* < 0.03). Lastly, McCoy and colleagues [48] evaluated an intervention consisting of a real-time pharmacy surveillance and a CDSS to reduce hospital-acquired ADEs in the setting of acute kidney injury (AKI). They found that the pharmacy surveillance on top of a CDSS had no significant effect on AKI-related potential ADEs or actual ADEs (*p* = 0.4). None of these studies measured uADEs as an outcome, and two did not assess the preventability of ADEs; i.e. pADEs [43, 47].

Lack of statistical power [43, 48], no assessment of ADE preventability [43, 47], as well as short exposure to the intervention [43, 44], may explain the absence of an effect on ADE incidence in study by Schmader and colleagues and McCoy and colleagues [43, 48], and only a modest reduction in ADEs in study by Trivalle and colleagues and Wehling and colleagues [44, 47]. O’Connor and colleagues [45] and O’Sullivan and colleagues [46] did not report an assessment of local ADEs and/or risk analyses with physicians as input for the chosen interventions, which may explain a lower impact of their intervention (48 and 34% reduction in hospital-acquired pADEs in O’Connor and O’Sullivan, respectively) in comparison to our intervention (51% reduction in hospital-acquired pADEs). Insights from implementation science show that involving front-line workers in the development of patient safety interventions (co-design) and using local insights about the extent and explanations for a patient safety problem at hand, are factors known to increase success rate of patient safety interventions in hospitals [49–51].

Although we found a significant reduction in hospital-acquired pADEs and uADEs, prescribing errors resulting in under- and overtreatment appeared to be least affected by our intervention. Also, recommendations regarding a lack of clear indication and an inappropriate choice of medication for an indication were least accepted. These are considered major problems in (de-)prescribing medication for older patients [52]. The resistance of these problems to our intervention may be explained by several factors. First, Dutch hospital pharmacists are not part of the medical team and are, probably, less versed in pharmacotherapy than clinical pharmacists in other countries [27–29]. This may have reduced the acceptance of their recommendations. Second, changing chronically used medication requires considerable effort (communication with patients and other care professionals, effect re-evaluation [53, 54]) for a patient often admitted to the hospital for only a short period of time. This makes medical residents reluctant to introduce these changes [5, 53]. Also, the errors of under- and overtreatment can be viewed as a strategic type of error, where the advice of hospital pharmacists pointed at problems of uncertainty about whether or not to (de)prescribe a treatment. In contrast, the errors of contra-indication, dosing errors or inappropriate choice errors can be viewed as a tactical type of error, where the advices of pharmacists point at prescribing decision already made. Apparently, medication errors of the strategic type are more challenging to reduce in comparison to errors of the tactical type.

### Strengths & limitations

Here we would like to highlight four aspects of this study that should be taken into consideration when assessing its strengths and limitations: a) the ITS design in light of practical constrains and new insights, b) ADE assessment by experts, c) the clinical relevance of the findings in light of the delay in publishing this manuscript, and d) the generalizability in light of the intervention being tailored to the Dutch healthcare setting.

#### ITS design

By following the EPOC criteria for short ITS studies regarding the number of data points and the number of observations per data point [33], we were able to assess the occurrence of trends which could have influenced our outcome measures. This is of particular importance given the time elapsed between end of the baseline and the start of the intervention period. No pre- or post-intervention trends were detected. However, new insights about ITS methodology show that, for an accurate estimation of secular trends, autocorrelation and seasonality, more sampling points are required [55, 56]. Furthermore, in retrospect, procedures for sample size and power calculation for ITS studies as recently proposed by Hawley et al. [57], would have been valuable for this study. On the other hand, even with more sampling points, the ITS study design precludes a straightforward attribution of causation. Therefore, although the ADE assessment process in the present study was reliable, and outcome models were adjusted, alternative explanations for the differences in the rates of hospital-acquired pADEs and uADEs cannot be ruled out. The two-year gap between the end of the baseline measurement period (November 2007) and the start of the intervention measurement period (November 2009) in this study might have had some influence. A parallel control group would have substantially improved the comparability with the intervention period. Unfortunately, in our setting, such a group was very hard to identify [35]. Also, an ITS analysis works by projecting the trend in the pre-intervention period onto the post-intervention period, thus providing a counterfactual to compare with the observed trend. A large gap between the periods may reduce the validity of such a comparison. To circumvent this limitation, we included ADRs as an internal validity measure, because of their non-responsiveness, by definition, to any intervention. The rate of ADRs per 100 hospitalizations remained constant throughout the whole study period (difference 0.8, 95% CI: − 8.4 to 10.0, *P* = 0.86). Another remedy comes from the fact that the reduction in event types (Fig. 1) corresponds with the types of recommendations made by the hospital pharmacists (Additional file 7), which also validates our results. In addition, we interviewed patient safety officers and reviewed annual management reports of the participating hospitals, and found that no interventions aiming at improving safety of prescribing in older inpatients have been implemented during the two-year gap. A higher awareness of physicians as a result of national and international patient safety campaigns is conceivable, although a significant reduction in preventable patient harm, only by higher awareness, is very unlikely [21, 58].

#### ADE assessment by experts

The two-year gap between the end of the baseline measurement period and the start of the intervention measurement period is explained by the comprehensive character of our ADE identification and assessment strategy [19], which required an estimated 1 hour per patient for chart abstraction and 2 hours per patient for patient chart review. Recruiting pharmacy students and research nurses for chart abstractions, training them appropriately, and developing handbooks and forms, was a time-consuming endeavor. The experts eventually appointed were only available part-time, as well as the pharmacy students and research nurses.

#### Clinical relevance

The results presented in this manuscript describe an investigation started in 2007 and completed in 2010. The publication delay was caused by personal circumstances of one of the authors. Since 2010, only a few publications, discussed above [43–48], reported about the effect of a medication review on hospital-acquired pADEs in older inpatients. In addition, to the best of our knowledge, this study is the first presenting an evaluation of the effect of such intervention on timely recognition of ADEs in older inpatients (uADEs). Furthermore, we feel that our study is still relevant, because medication prescribing (selecting, informing patients, initiating, monitoring and continuation) to older people continues providing major challenges to many physicians [5].

#### Generalizability

The generalizability of our results may be less in hospitals employing more pharmacists per bed than in The Netherlands [27]. As a result of this, standard of care practices in such hospitals involve more pharmacists in ADE recognition and prevention, which may reduce the impact of our intervention, if it were applied there. We found *grosso modo* the same classes of drugs implicated in preventable ADEs as the authors of studies discussed earlier. This shows that there are some similarities between the Dutch healthcare and research setting and other settings. Notwithstanding the Dutch context, we believe that our study provides a number of general learning points to consider when developing and evaluating prescribing safety intervention, like: measuring clinical outcomes causally linked to prescribing safety like pADEs and uADEs, assessing specific prescribing safety problems at a local level before deciding on the intervention strategy, and involving physicians and pharmacists (especially those in training) in conceiving prescribing safety interventions.

### Implications for future research

To address inappropriate prescribing in older inpatients, a patient-centered multidisciplinary team approach is needed [59]. Based on the results of this study, participation of hospital pharmacists in such teams is recommended. To enable hospital pharmacists to engage in such on-ward activities in an efficient and effective manner (especially in settings with limited hospital pharmacist staffing), the development and use of computerized tools, which could help to distil medication related problems specific for the geriatric patient population, should be aimed for [60]. Additionally, innovative methods from machine learning hold promise to optimize ADE detection by reusing data registered in electronic hospital records [61]. Machine learning algorithms for ADE detection could serve as an efficient and more real-time alternative to the time-consuming and often retrospective manual patient chart review. Also, the generalizability of our findings to other patient populations remains to be investigated. Future ITS studies should consider sample size and power calculation recommended by Hawley et al. [57] to avoid carrying out underpowered studies, and, if feasible, include a higher number of sampling points [55, 56].

## Conclusions

In conclusion, a comprehensive and structured medication review by hospital pharmacists, followed by a face-to-face feedback to the physicians on the ward, can significantly improve safety of medication prescribing in older inpatients. Tailoring the intervention strategy to local ADE data, resources and needs of internal medicine residents, may all have contributed to the significant reduction in hospital-acquired pADEs and uADEs.

## Supplementary Information

**Additional file 1:** SQUIRE Checklist.

**Additional file 2:** TIDieR checklist.

**Additional file 3:** Trends in hospital-acquired pADEs and uADEs.

**Additional file 4:** Global ITS models.

**Additional file 5:** Univariate analyses.

**Additional file 6:** Medication errors.

**Additional file 7:** Acceptance of hospital pharmacist recommendations.

## Data Availability

The datasets generated and/or analyzed during the current study are not publicly available due to ethical and legal restrictions, given that data contain potentially identifying information. For this study we collected data at our institution and two other hospitals, and we do not have the rights to share data of these two other hospitals. Data collected at our institution are available with permission by the Head of Clinical Research Unit of the Amsterdam University Medical Center, in consultation with the first author for researchers who meet the criteria for access to confidential data.

## Abbreviations

ADE: Adverse Drug Event
pADE: preventable Adverse Drug Event
uADE: unrecognized Adverse Drug Event
ADR: Adverse Drug Reaction
CAREFUL: pharmacist Coordinated ADE Reducing Efforts For Use in all Levels of healthcare
CDSS: Clinical Decision Support System
CPOE: computerized physician order entry
CTCAEv3: Common Terminology Criteria for Adverse Events version 3.0
DRP: drug-related problem
EPOC: Cochrane Effective Practice and Organization of Care Review Group
FORTA: Fit FOr The Aged
INR: international normalization ratio
ITS: interrupted time series
START: Screening Tool to Alert to Right Treatment
STOPP: Screening Tool of Older Persons’ Prescriptions
WHO: World Health Organization
WINGS: Ward-oriented pharmacy In Newly admitted Geriatric Seniors
WMO: the Dutch Medical Research Human Subjects Act
ZonMW: The Netherlands Organization for Health Research and Development
