# Supplementary material for: The effect of structured medication review followed by face-to-face feedback to prescribers on adverse drug events recognition and prevention in older inpatients – a multicenter interrupted time series study
Source: BMC Geriatr. 2022 Jun 17;22:505. doi: 10.1186/s12877-022-03118-z (PMC9206349; doi:10.1186/s12877-022-03118-z)
Supplement: Supplementary file 2 — Additional file 2: TIDieR checklist. [file 12877_2022_3118_MOESM2_ESM.pdf]

TIDieR
Template for Intervention Description and Replication

The TIDieR (Template for Intervention Description and Replication) Checklist*:

Information to include when describing an intervention and the location of the information

Item number	Item
1.	**BRIEF NAME**

Provide the name or a phrase that describes the intervention.

Structured medication review by hospital pharmacists, followed by face-to-face feedback to prescribers.

2. WHY

Describe any rationale, theory, or goal of the elements essential to the intervention.

As the first step in developing the intervention, multidisciplinary meetings were organized at each participating hospital, where a delegation of 5 to 10 members of internal medicine staff and residents, and hospital pharmacy staff took part. During these meetings, the Bow-Tie model was used to structure the discussion. The Bow-Tie approach combines causes, errors, preventive and recovery measures, and consequences in a single model. Furthermore, it gives insight into the magnitude and causes of existing safety risks.

Internal medicine residents pinpointed two unsafe practices in the process of prescribing. These two unsafe practices were, in their opinion, most likely related to the high occurrence of hospital-acquired pADEs and suboptimal ADE recognition in older inpatients:

1) On the professional level: in the participating hospitals, the daily care of patients was provided by junior medical residents, who had one to two years of clinical experience and were supervised by attending senior physicians. Gaps in geriatric pharmacotherapy knowledge and skills were felt to be of major concern across all care settings and levels of medical experience.

2) On the organizational level: in The Netherlands (as well as in most European countries), hospital pharmacists provide only limited supervision on prescribing and are not part of medical teams on the wards. The usual practice is a daily on-call availability for medication-related problems and daily screening of medication alerts overridden by physicians during prescribing.

Therefore, to improve safety of the prescribing process, the internal medicine residents proposed a regular medication review by a hospital pharmacist, followed (on the same day) by face-to-face feedback on prescribing on the ward to the physician involved. Although the effectiveness of medication review by hospital pharmacists on clinical endpoint remains unclear and underinvestigated, such an intervention does appear beneficial in terms of reducing inappropriate prescribing and prescribing errors.

The intervention ultimately implemented consisted of a medication review and face-to-face feedback on prescribing by a hospital pharmacist on average three days per week.

3. **WHAT: MATERIALS**

Describe any physical or informational materials used in the intervention, including those provided to participants or used in intervention delivery or in training of intervention providers. Provide information on where the materials can be accessed (e.g. online appendix, URL).

Internal medicine residents: a pocket-sized card with an overview of most frequent Drug Related Problems (DRPs) in older inpatients was disseminated to all residents working in Internal Medicine wards of the participating hospitals. Also, all and newly started internal medicine residents were provided an educational session about geriatric pharmacotherapy.

Hospital pharmacists: All (potential) DRPs identified, together with the recommendations to resolve these, were registered on a standardized consultation form. The hospital pharmacists participating in the intervention were educated by the project leader (JK) about the findings from the baseline measurement and about the procedure of medication review and registrations to be conducted. A written instructions on how to provide the intervention was provided. Also, clinical guidelines and recent literature with relation to the DRPs identified was bundled in a folder for easy access during medication review.

All materials (in Dutch) are available upon request from the first author.

4. **WHAT: PROCEDURES**

Describe each of the procedures, activities, and/or processes used in the intervention, including any enabling or support activities.

Medication review: Patient and drug charts were screened for the following (potential) drug-related problems (DRPs): discrepancies between hospital and home medication use, side-effects, under- and overprescribing, under- and overdosing (including renal and liver function related dosing adjustments), (relative) contra-indications, drug-drug interactions, and lack of insufficient monitoring of laboratory values, such as electrolytes, renal and liver function, and the international normalization ratio (INR). All (potential) DRPs identified, together with the recommendations to resolve these, were registered on a standardized consultation form. Subsequently, the results of the medication review were discussed face-to-face with the internal medicine residents on the wards. Such face-to-face discussions facilitated the exchange of knowledge and any additional information about the patient’s condition. The hospital pharmacist recorded whether proposed recommendations were accepted by the residents or not. Any changes to pharmacotherapy or clinical status of patients already reviewed were also monitored during patients’ hospital stay.

Supporting activities: To introduce the intervention to all and newly starting internal medicine residents, at each participating hospital, an educational session about geriatric pharmacotherapy was provided in October 2009 and April 2010 (standard starting moments of internal medicine rotations). During these sessions, the study was introduced, example cases from the baseline measurement were

discussed, and the intervention explained. Also, a pocket-sized card with an overview of most frequent DRPs in older inpatients (as identified during the baseline measurement), to be used as a mnemonic, was disseminated to all residents working in Internal Medicine wards of the participating hospitals. A newsletter about the study to all internal medicine and hospital pharmacy staff of the participating hospitals was provided at the start of the intervention period.

5. WHO PROVIDED

For each category of intervention provider (e.g. psychologist, nursing assistant), describe their expertise, background and any specific training given.

The medication review with face-to-face feedback was provided by hospital pharmacists. In case of staff shortage, senior hospital pharmacy residents were also involved (after being trained by the project leader and supervised by hospital pharmacists). Pharmacy technicians supported the hospital pharmacists (residents) in gathering overviews of home medications and in medication verification, as well as in conducting easy checks such as: are laxatives prescribed in combination with opiates, and is gastric protection prescribed in combination with NSAIDs.

6. HOW

Describe the modes of delivery (e.g. face-to-face or by some other mechanism, such as internet or telephone) of the intervention and whether it was provided individually or in a group.

Face-to-face to individual prescribers.

7. WHERE

Describe the type(s) of location(s) where the intervention occurred, including any necessary infrastructure or relevant features.

Three hospitals in the Netherlands. In The Netherlands (as well as in most European countries), hospital pharmacists provide only limited supervision on prescribing and are not part of medical teams on the wards. The usual practice is a daily on-call availability for medication-related problems and daily screening of medication alerts overridden by physicians during prescribing. In all participating hospitals, computerized physician order entry (CPOE) systems were operational at the time of this study. All CPOE systems in Dutch hospitals use the Dutch drug database ‘G-Standard’ which contains safety information on all drugs registered in The Netherlands. Based on the safety information in the G-Standard, the CPOE systems generate drug-drug interaction warnings, duplicate orders alerts, and general dosing advice, i.e. basic clinical decision support. When a physician enters a medication order in this type of CPOE, alerts are intrusively shown on the screen. Overridden alerts are logged for hospital pharmacy review. This review is usually conducted retrospectively (within 24 hours after ordering) by a hospital pharmacist once a day. This process was the only routine medication review step by hospital pharmacists at the time of this study.

This is different from countries such as the United Kingdom (UK) and the United States (US) of America, employing more pharmacists per bed, provide pharmaceutical care under the denominator “clinical pharmacy”. Clinical pharmacy involves pharmacists in daily rounding teams, many of them becoming specialists in the pharmacotherapy field that is applied most frequently in their ward, and they often are licensed to prescribe medication. Pharmacists in most European countries, except the UK, are less numerous, and tend to be generalists with broader, but more superficial, pharmacotherapy expertise.

8. **WHEN and HOW MUCH**

Describe the number of times the intervention was delivered and over what period of time including the number of sessions, their schedule, and their duration, intensity or dose.

The medication reviews with face-to-face feedback were provided on average three days per week. Such a frequency was considered achievable in terms of hospital pharmacy staff allocation and time investment per patient. On review days, the appointed hospital pharmacist conducted a structured medication review of patients admitted on the review day and on in-between review days.

9. **TAILORING**

If the intervention was planned to be personalised, titrated or adapted, then describe what, why, when, and how.

In case of staff shortages, hospital pharmacist residents could participate in the intervention. Also, the amount of support by pharmacy technicians in providing easy checks could be adapted per participating site according to local staffing possibilities.

10.‡ **MODIFICATIONS**

If the intervention was modified during the course of the study, describe the changes (what, why, when, and how).

In case of staff shortages, senior hospital pharmacist residents were allowed to provide the intervention.

11. **HOW WELL**

Planned: If intervention adherence or fidelity was assessed, describe how and by whom, and if any strategies were used to maintain or improve fidelity, describe them.

The project leader was in contact on regular basis with the hospital pharmacy departments providing the intervention. At each site a dedicated hospital pharmacist and internal medicine senior physician were appointed to monitor the study progress and intervention provided. At each site, a written instructions and supporting materials (literature folder) were provided to the hospital pharmacy (residents) providing the intervention. Furthermore, site visits by the project leader were conducted each one to one-and-half month. During these site visits the project leader met with the appointed staff members of the hospital pharmacy and internal medicine wards to discuss any issues with the intervention and resolve them if applicable.

**12.‡**	Actual: If intervention adherence or fidelity was assessed, describe the extent to which the intervention was delivered as planned.<br><br>The main issue reported during the intervention period was shortages of hospital pharmacists to provide the intervention. This issue was resolved by allowing to involve senior hospital pharmacy residents in case of shortages. Also, hospital pharmacy technicians were involved to support hospital pharmacists (residents) in conducting easy checks as preparation for the medication reviews. Furthermore, finding the best time for face-to-face contact with the internal medicine residents was challenging. Especially at times when patient per physician load was high. Overall, the best time was after hand-overs and patient rounds were finalized in the morning.

† If the information is not provided in the primary paper, give details of where this information is available. This may include locations such as a published protocol or other published papers (provide citation details) or a website (provide the URL).
‡ If completing the TIDieR checklist for a protocol, these items are not relevant to the protocol and cannot be described until the study is complete.

* We strongly recommend using this checklist in conjunction with the TIDieR guide (see BMJ 2014;348:g1687) which contains an explanation and elaboration for each item.
