# Supplementary material for: The effect of structured medication review followed by face-to-face feedback to prescribers on adverse drug events recognition and prevention in older inpatients – a multicenter interrupted time series study
Source: BMC Geriatr. 2022 Jun 17;22:505. doi: 10.1186/s12877-022-03118-z (PMC9206349; doi:10.1186/s12877-022-03118-z)
Supplement: Supplementary file 3 — Additional file 3: Trends in hospital-acquired pADEs and uADEs. [file 12877_2022_3118_MOESM3_ESM.pdf]

**Additional file 3:** Trends over time in hospital-acquired pADEs and in uADEs.

We conducted a bootstrapping procedure (with replacement and stratified on sampling point and hospital according to our sampling procedure showed in the third figure) and used the accelerated bias-corrected percentile limits method to calculate 95% confidence levels as recommended by Puth et al. J Anim Ecol 2015;84: 892-897. This resulted in the following graphical trend representation for hospital-acquired pADEs and for uADEs showing mean with lower and upper limits of 95% CI for each sampling point.

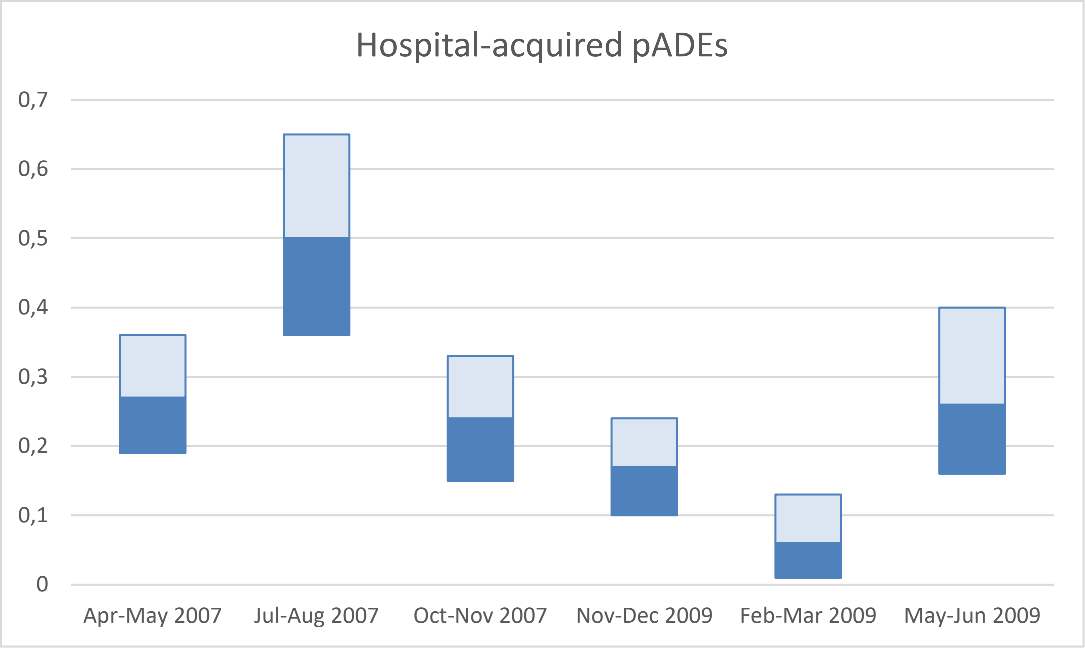


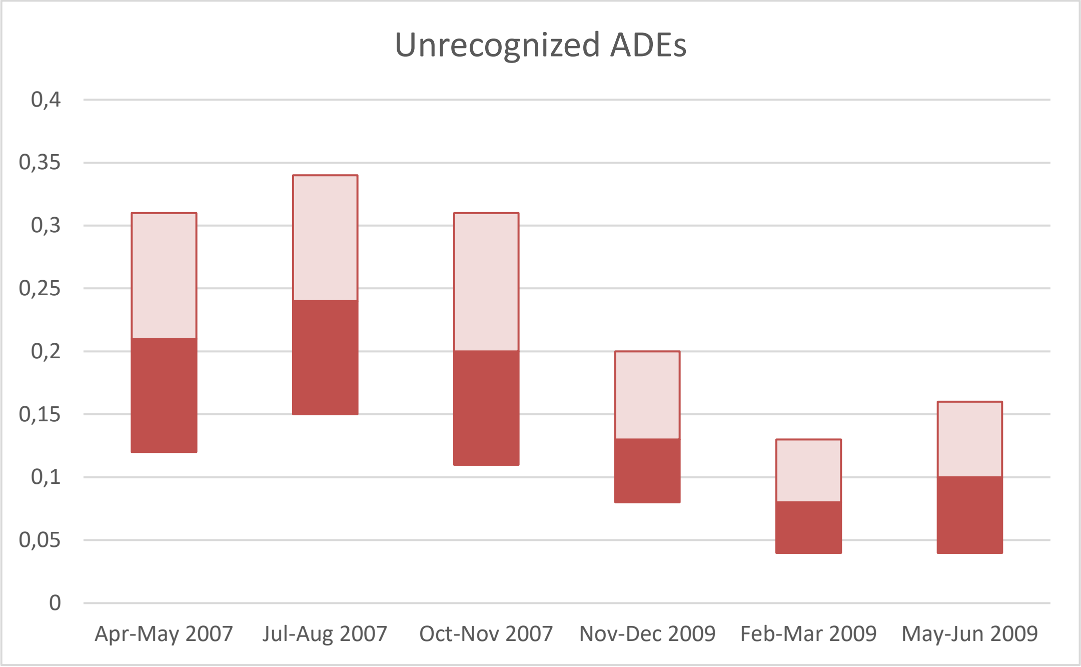

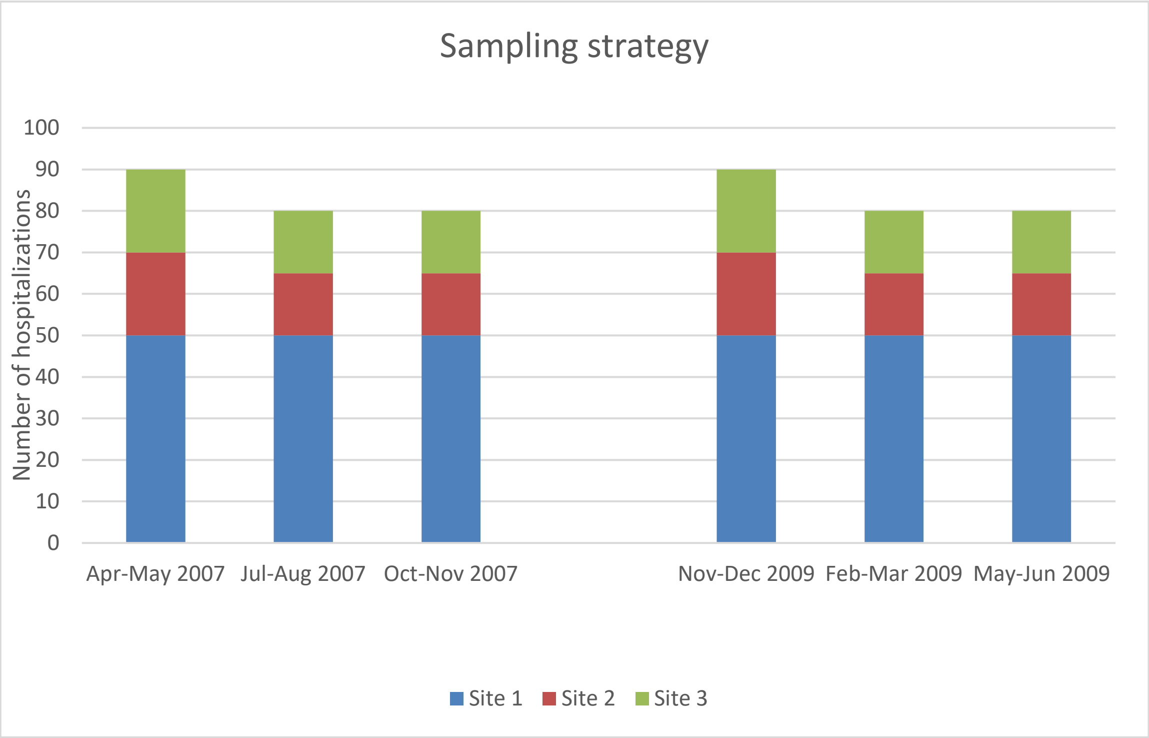
Sampling strategy
Number of hospitalizations
100
90
80
70
60
50
40
30
20
10
0
Apr-May 2007
Jul-Aug 2007
Oct-Nov 2007
Nov-Dec 2009
Feb-Mar 2009
May-Jun 2009
Site 1
Site 2
Site 3
