# Supplementary material for: The effect of structured medication review followed by face-to-face feedback to prescribers on adverse drug events recognition and prevention in older inpatients – a multicenter interrupted time series study
Source: BMC Geriatr. 2022 Jun 17;22:505. doi: 10.1186/s12877-022-03118-z (PMC9206349; doi:10.1186/s12877-022-03118-z)
Supplement: Supplementary file 4 — Additional file 4: Global ITS models. [file 12877_2022_3118_MOESM4_ESM.pdf]

Additional file 4: Global Interrupted Time Series (ITS) models for hospital-acquired preventable ADEs (pADEs) and unrecognized ADEs (uADEs).

**ITS model for hospital-acquired pADEs**		
**ITS parameter**	**Exp(B) (95% CI)**	**P value**
**Intercept**	0.35 (0.20 – 0.61)	< 0.001
**Pre-intervention trend**	0.97 (0.74 – 1.26)	0.823
**Post-intervention trend**	1.36 (0.86 – 2.15)	0.190
**Change in level post-intervention**		
Intervention period	0.29 (0.11 – 0.73)	0.009
Baseline period	*Reference category*	
**ITS model for uADEs**		
**ITS parameter**	**Exp(B) (95% CI)**	**P value**
**Intercept**	0.23 (0.11 – 0.45)	< 0.001
**Pre-intervention trend**	0.98 (0.71 – 1.35)	0.890
**Post-intervention trend**	0.86 (0.49 – 1.53)	0.616
**Change in level post-intervention**		
Intervention period	0.68 (0.24 – 1.94)	0.472
Baseline period	*Reference category*	

Hospital-acquired pADEs: Before the intervention, there was no significant trend over consecutive samples in incidence of hospital-acquired pADEs (p-value = 0.82). The intervention itself was associated with a reduced incidence (p-value = 0.009). No post-intervention trend in incidence was observed (p-value = 0.19). Because only a shift in incidence by introduction of the intervention was observed and no further trends seemed present, we used only the change in level post-intervention in the subsequent multivariate analyses (the most parsimonious model).

uADEs: Trends in incidences of unrecognized ADEs before (p-value = 0.89) or after (p-value = 0.62) the intervention were not observed and only the main result reflecting the absence (p-value = 0.47) of impact by the intervention on the incidence of unrecognized ADEs was included in subsequent multivariate analyses (the most parsimonious model).
