# Supplementary material for: The effect of structured medication review followed by face-to-face feedback to prescribers on adverse drug events recognition and prevention in older inpatients – a multicenter interrupted time series study
Source: BMC Geriatr. 2022 Jun 17;22:505. doi: 10.1186/s12877-022-03118-z (PMC9206349; doi:10.1186/s12877-022-03118-z)
Supplement: Supplementary file 5 — Additional file 5: Univariate analyses. [file 12877_2022_3118_MOESM5_ESM.pdf]

Additional file 5: Univariate analyses outputs. Explanatory variables that are significant predictors (p-value ≤ 0.1) in the univariate analysis were included in the subsequent multivariate regression models.

**Hospital-acquired preventable ADEs**		
	**OR (95% CI)**	**P value**
**ITS parameter**		
**Change in level post-intervention**		
Intervention period	0.494 (0.340 – 0.718)	< 0.001
Baseline period	*reference category*	
**Patient characteristics**		
**Age**	1.008 (0.985 – 1.031)	0.501
**Sex**		
Female	1.117 (0.786 – 1.588)	0.538
Male	*reference category*	
**Home situation**		
Living independently	0.821 (0.509 – 1.326)	0.421
Living non-independently	*reference category*	
**Type of admission**		
Acute	0.580 (0.374 – 0.900)	**0.015**
Elective	*reference category*	
**Time of admission**		
Weekday	0.761 (0.450 – 1.287)	0.309
Weekend	*reference category*	
**Length of stay**	Excluded[a]	
**Number of preadmission medications**	1.048 (0.998 – 1.100)	**0.062**
**Number of hospital medications**	1.070 (1.034 – 1.108)	**< 0.001**
**Charlson Co-morbidity Index score**	1.065 (0.975 – 1.164)	**0.162**[b]
**Number of concomitant diseases**	1.023 (0.931 – 1.124)	0.637
**Cognitive impairment on admission**		
No	0.769 (0.477 – 1.241)	0.283
Yes	*reference category*	

**MDRD *e*GFR (ml/min/1.73 m^2)**		
≥ 60	1.675 (1.055 – 2.660)	**0.029**
30-59	1.543 (1.006 – 2.369)	**0.047**
≤ 29	*reference category*	

[a] We have excluded length of stay (LOS) because a longer LOS can be a result of hospital-acquired pADE and at the same time a longer LOS is also a risk-factor for hospital-acquired pADE.
[b] This p-value was assessed as “borderline” and therefore this variable was included in the multivariate analysis.

Unrecognized ADEs			
		**OR (95% CI)**	**P value**
**ITS parameter**			
	Intervention period	0.481 (0.302 – 0.769)	0.002
	Baseline period	*reference category*	
**Patient characteristics**			
**Age**		1.008 (0.985 – 1.031)	0.501
**Sex**			
	Female	1.027 (0.659 – 1.602)	0.905
	Male	*reference category*	
**Living independently**			
	Living independently	0.953 (0.579 – 1.786)	0.953
	Living non-independently	*reference category*	
**Type of admission**			
	Acute	0.997 (0.513 – 1.936)	0.992
	Elective	*reference category*	
**Time of admission**			
	Weekday	1.124 (0.63 – 2.004)	0.692
	Weekend	*reference category*	
**Length of stay**		Excluded[a]	
**Number of preadmission medications**		1.073 (1.010 – 1.140)	**0.023**
**Number of hospital medications**		0.993 (0.946 – 1.042)	0.767
**Charlson Co-morbidity Index score**		0.887 (0.784 – 1.003)	**0.057**
**Number of concomitant diseases**		1.035 (0.919 – 1.165)	0.573
**Cognitive impairment on admission**			
	No	1.379 (0.830 – 2.293)	0.215
	Yes	*reference category*	
**MDRD *e*GFR[c] (ml/min/1.73 m^2)**			
	≥ 60	1.056 (0.582 – 1.918)	0.857

	30-59	1.270 (0.764 – 2.112)	0.357
	≤ 29	*reference category*	

[a] We have excluded length of stay (LOS) because a longer LOS can be a result of hospital-acquired pADE and at the same time a longer LOS is also a risk-factor for hospital-acquired pADE.
