# Supplementary material for: The effect of structured medication review followed by face-to-face feedback to prescribers on adverse drug events recognition and prevention in older inpatients – a multicenter interrupted time series study
Source: BMC Geriatr. 2022 Jun 17;22:505. doi: 10.1186/s12877-022-03118-z (PMC9206349; doi:10.1186/s12877-022-03118-z)
Supplement: Supplementary file 6 — Additional file 6: Medication errors. [file 12877_2022_3118_MOESM6_ESM.pdf]

Additional file 6. Types of medication errors for the baseline versus the intervention period.

**Medication error**	**Baseline**	**Intervention**	**Most frequently involved medication**
	No. (%)	No. (%)	
	n = 83	n = 41	
**Prescribing error**	73 (88.0)	36 (88.0)	
Contra-indicated medication	20	5	Oral antidiabetics (5), Diuretics (5), Statins (3), RAAS inhibitors (2), Narcotics (2), [illegible]
Undertreatment (omission)	19	16	Omission of iron preparations (11), laxatives (5), diuretics (4), corticosteroids (3), insulin
Dosing errors	14	5	Antibiotics (6), Digoxin (2), Calcium channel blockers (1), Insulin (1), Statins (1)
Inappropriate choice (drug or route of	11	5	Antibiotics (14), beta-blocker (1), Digoxin (1)
Drug-drug interactions	6	2	Coumarin (7), Antibiotics (1)
Overtreatment	2	3	Antibiotics (1), Insulin (1), Beta-blockers (1),
Drug duplication	1	0	Oral antidiabetics (1)
**Medication administration error**	5 (6.0)	3 (7.3)	Antibiotics (1), Coumarin (2), Insulin (2), Digoxin (1), Beta-blocker (1)
**Monitoring error**	5 (6.0)	2 (4.9)	Antibiotics (4), Diuretics (1)

pADEs, preventable adverse drug events. Abbreviations: RAAS, Renin-Angiotensin-Aldosterone-System
