# Supplementary material for: The effect of structured medication review followed by face-to-face feedback to prescribers on adverse drug events recognition and prevention in older inpatients – a multicenter interrupted time series study
Source: BMC Geriatr. 2022 Jun 17;22:505. doi: 10.1186/s12877-022-03118-z (PMC9206349; doi:10.1186/s12877-022-03118-z)
Supplement: Supplementary file 7 — Additional file 7: Acceptance of hospital pharmacist recommendations. [file 12877_2022_3118_MOESM7_ESM.pdf]

Additional file 7. Acceptance of hospital pharmacist recommendations formulated based on results from the structured medication reviews.

**Recommendation**	**Number of recommendations (% accepted)**
Omission of prophylactic (co)-medication	96 (66.7)
Potential discrepancies between hospital and home medication use	78 (55.1)
Dosing or contra-indication according to renal or liver function	56 (64.3)
No clear indication, reconsider discontinuation of drug	36 (38.9)
Drug-drug interactions	22 (91.0)
Electrolytes imbalances due to medication	21 (66.7)
Hypertension over- and undertreatment	21 (76.2)
Monitoring of INR in patients with known drug-drug interactions with coumarin	16 (75.0)
Restrict 'as needed' and overall use of benzodiazepines and pain medications	15 (80.0)
Inappropriate choice of medication for an indication	12 (8.3)
Other	27 (51.9)
**Total**	**400 (61.5)**

RAAS, Renin-Angiotensin-Aldosterone-System; NSAIDs, Non-Steroidal Anti-Inflammatory Drugs
